# ORPHANED ELEPHANTS EXHIBIT HIGHER THYROID HORMONE CONCENTRATIONS COMPARED TO CONTROLS

**DOI:** 10.1093/geroni/igad104.3512

**Published:** 2023-12-21

**Authors:** Daniella Chusyd, Janine Brown, Steve Paris, Stephanie Dickinson, Tessa Steiniche, Steven Austad, David Allison, Michael Wasserman

**Affiliations:** Indiana University, Bloomington, Indiana, United States; Smithsonian Conservation Biology Institute, Front Royal, Virginia, United States; Smithsonian Conservation Biology Institute, Front Royal, Virginia, United States; Indiana University, Bloomington, Indiana, United States; Indiana University, Bloomington, Indiana, United States; University of Alabama at Birmingham, Birmingham, Alabama, United States; Indiana University, Bloomington, Indiana, United States; Indiana University, Bloomington, Indiana, United States

## Abstract

Wild elephant populations provide a natural experiment to study biological effects of early adverse events, such as being orphaned due to poaching. We compared fecal glucocorticoid metabolite (fGCM) and thyroid hormone (T3) concentrations, and body condition between rescued orphaned elephants and similarly age-matched, control wild elephants. In 2021, single fecal samples were collected during the early (n=20 orphans; n=58 controls) and late (n=20 orphans; n=22 controls) dry seasons. Age was known (+/- 3 months) for orphans and estimated by dung diameter (+/- 1 year) for controls. Sex was assigned based on morphology and unknown for some controls. Body condition scores (BCS: 1 to 9) were assigned at time of sample collection. Linear Mixed Models accounting for repeated measures were performed adjusted for age and season. There was no difference in fGCM between orphans (mean 101.48, SD 59.62ng/g) and controls (mean 93.86, SD 49.97ng/g) (p=0.213). Orphans (mean 271.75, SD 123.16ng/g) had higher T3 concentrations compared to controls (mean 98.65, SD 71.89ng/g) (p< 0.001). There was no difference in BCS between groups (p=0.629). In orphans, fGCM was associated with age (beta=-0.499, p=0.011). In controls, T3 was correlated with fGCM (beta=0.287, p=0.011) and season was associated with fGCM (p=0.008) and BCS (p=0.044). Either because of living under human care or because of alterations in T3 secretion, seasonal factors do not seem to impact orphans’ health status to the same extent as in control elephants. The support of an adopted herd may attenuate the physiological stress associated with experiencing trauma in our study population.

